# An Explorative Study of the Causal Pathogenesis of Green Liver Discoloration in Organically Reared Female Bronze Turkeys (*Meleagris gallopavo*) Considering the Infectious Risk Factors

**DOI:** 10.3390/ani13050918

**Published:** 2023-03-03

**Authors:** Larissa Cuta, Christoph Georg Baums, Kerstin Cramer, Maxi Harzer, Jutta Hauptmann, Kristin Heenemann, Maria-Elisabeth Krautwald-Junghanns, Ines Stegmaier, Thomas W. Vahlenkamp, Volker Schmidt

**Affiliations:** 1 Clinic for Birds and Reptiles, Faculty of Veterinary Medicine, University of Leipzig, An den Tierkliniken 17, 04103 Leipzig, Germany; 2 Institute of Bacteriology and Mycology, Faculty of Veterinary Medicine, University Leipzig, An den Tierkliniken 29, 04103 Leipzig, Germany; 3 Institute of Virology, Faculty of Veterinary Medicine, University of Leipzig, An den Tierkliniken 29, 04103 Leipzig, Germany

**Keywords:** Turkey Osteomyelitis Complex, green liver, hemorrhagic enteritis virus, aseptic bone necrosis

## Abstract

**Simple Summary:**

Organically raised turkeys investigated in a recent study showed a high prevalence of green liver discoloration. The condition is commonly associated with bone alterations and is potentially caused by opportunistic bacteria. This study was performed to determine possible infectious risk factors and reduce disease prevalence. The prevalence of green livers has decreased between both studies, and there was no explicable significant correlation with bacterial or parasitological findings. However, there was a significant correlation between green livers and the immunosuppressive turkey hemorrhagic enteritis virus at the early fattening stage. Hens with virus detection had impaired physical health. At the late fattening stage, there was a significant correlation between green livers and joint/bone lesions, as described in previous literature. It can be assumed that the hemorrhagic enteritis virus affects the pathogenesis of green liver discoloration at the early fattening stage. Therefore, an adequate vaccination schedule should be implemented in poultry farms to reduce the prevalence of discoloration and improve animal health. However, further standardized investigations to determine and evaluate possible infectious risks regarding green liver discoloration are necessary.

**Abstract:**

A recent study revealed that organically raised Bronze turkeys showed a high prevalence of green liver discoloration. This alteration is commonly associated with the Turkey Osteomyelitis Complex and potentially caused by opportunistic bacteria. Therefore, 360 organically fattened Bronze turkeys were examined post-mortem throughout two fattening trials with two examinations each to determine possible infectious risk factors and reduce disease prevalence. Clinical and pathoanatomical examinations were performed on every hen. Histopathological, bacteriological, parasitological, and virological examinations were performed on at least six hens without and, if applicable, six hens with green livers on each examination date. Overall, 9.0% of all hens had a green liver without a correlation with bacterial or parasitological findings but multiple health impairments. The discoloration correlated significantly with the detection of immunosuppressive turkey hemorrhagic enteritis virus at the early stage and macro- and histological joint/bone lesions at the late fattening stage, indicating the presence of two different predisposing pathogeneses. Flocks not being vaccinated against hemorrhagic enteritis but having a virus-positive sample showed the highest prevalence of green liver discoloration and developed worse in various parameters. In conclusion, an adequate vaccination schedule and the prevention of field infections may lead to a decreased risk of performance reduction and improved animal health.

## 1. Introduction

The organic farming associations and Council Regulation (EU) No 2018/848 of 20 May 2018 on organic production and labeling of organic products place special requirements on the organic rearing of turkeys in Germany [1]. This serves to improve animal welfare as well as climate and environmental protection. During extensive statistical surveys from 2007 to 2017, the health status of conventionally and organically raised turkeys in Germany was recorded [2,3,4]. The latest study revealed that organically raised turkeys (Kelly Broad Breasted Bronze [BBB]) showed an approximately nine times higher prevalence of green livers (GL) at processing compared to conventionally raised B.U.T. 6 (British United Turkeys). The prevalences of GL for organically raised Bronze toms and hens were 34.8% and 33.2%, versus 3.8% and 4.4% for conventionally raised B.U.T. 6 toms and hens, respectively [2,4,5].

The pathogenesis of GL is mainly attributable to the accumulation of inter- and intrahepatic endogenous biliverdin [6]. This bile pigment represents a degradation product of erythrocytes. It is particularly relevant in birds due to the absence of the enzyme biliverdin reductase, which converts biliverdin to bilirubin [7]. Besides bacterial infection, other possible causes for the accumulation of biliverdin are increased hemolysis due to mechanical trauma, intoxication, hepatitis, necrosis, or obstructive outflow disorders [8]. Turkey osteomyelitis complex (TOC) is a disease predominantly described in conventionally fattened turkeys. It is commonly associated with GL and bone alterations, leading to economic losses due to the discarding of complete or partial carcasses [9]. The descriptions of this disease complex have many similarities with the findings from the predecessor study. A statistically significant correlation (*p* ≤ 0.05, r = 0.49) between prevalences of GL and joint pathologies was found at processing [4].

However, previous studies indicate that the alterations of TOC are associated with opportunistic bacteria like *Escherichia* (*E.*) *coli* or *Staphylococcus* (*S.*) *aureus* [10,11,12]. A further immunosuppressive effect of infection with the turkey hemorrhagic enteritis virus (HEV), leading to a higher prevalence of pathological findings associated with coliform bacteria, has been suggested [12]. Transient immunosuppression and related hemorrhagic enteritis are mediated by the release of proinflammatory cytokines as well as necrosis and apoptosis of primary target cells, B lymphocytes, and macrophages [13,14,15]. HEV belongs to the family *Adenoviridae*, genus *Siadenovirus*, and species *Turkey siadenovirus A*. It includes two strains, the avirulent turkey HEV and the virulent turkey adenovirus 3 [16]. It is a non-enveloped, linear, double-stranded DNA virus with an approximate genome length of 26.3 kb and is characterized by an open reading frame (ORF). The ORF encodes for the glycoside hydrolyzing enzyme sialidase [17,18,19]. Besides different bacteria, several other viruses and diseases in turkeys are known to affect the liver. These include, among others, *avian hepatitis E virus*, turkey viral hepatitis, aviadenoviruses, highly pathogenic avian influenza A viruses, and reoviruses [17,20,21,22,23,24,25,26,27].

This study was designed to identify associations between GL and infectious risk factors in organically raised turkeys. It was carried out to gain knowledge about the potentially infectious causative agents of GL disease in organic turkey farming. Further, it was conducted to implement strategies to avoid circumstances leading to discoloration and the associated TOC at the time of processing. The results may help to assess the importance of preventing HEV infection during an early stage of fattening to avoid economic losses and improve health and welfare.

## 2. Materials and Methods

The examinations did not require notification or approval, as in accordance with the German Animal Welfare Act (§ 7, paragraph 2, sentence 3).

### 2.1. Animals and Time Points of Examination

A total of five organic turkey farms fattening Bronze turkey hens of a Cartier genetic (including rearing farms) were selected for this investigation. In part, the selection was based on the selection criteria of high prevalence of GL at processing from the previous study [4,5]. This study included two consecutive trials, with the first examination trial extending over six months, starting in September 2020 and ending in March 2021. The second trial started in February 2021 and finished with the final examination in August 2021. As a consequence of avian influenza control measures in one farm’s area (flock No. 41, second examination) and quarantine measures in the scope of the SARS-CoV-2 pandemic (flock No. 31, second examination), two examinations were canceled. Due to the avian influenza epidemic in Germany in 2020/2021, flocks No. 21 (second examination trial) and No. 22 (first examination trial) were not granted any access to the outdoor enclosure. In Flock No. 11 (second examination trial), access to the outdoor enclosure became available during the end of the fattening phase. The turkeys of flock No. 41 were kept outside during the most part of the fattening period with variable access to woodland and a small stable (in the second trial, no access was allowed from week 11 onward). Recorded data of the farm workers of each farm considered in this investigation included the vaccination program, flock diseases and treatments, and daily mortality (Table S1). With respect to health protection measures during the SARS-CoV-2 pandemic, no personal attendance at the slaughterhouses was possible. Clinical and detailed post-mortem examinations were performed on 20 turkeys per flock between the 70th and 75th days of fattening (the first or early examination time) and the 120th to 127th days of fattening (the second or late examination time), respectively. In addition, an inspection of the turkeys was performed on the first days of rearing. The sample size was calculated based on an average prevalence of GL of 27.7% (Bronze- and B.U.T. 6/TP7/TP9-hens) in organically raised hens, with the lower limits of the 95% confidence intervals of 18.5% [4]. Using this lower limit with a maximum herd size of 2.500 turkeys [the maximum permissible number of fattening turkeys in one housing unit according to Regulation (EU) No. 2020/464 of 26 March 2020] and calculation according to Cannon and Roe, a sample size of n = 16 was required to demonstrate the presence of GL in organically raised hens [28]. Sample size was limited to 20 randomly drawn individuals per investigation time and flock. This can be explained by the fact that the sample, on the one hand, had to comprise a sufficient number of carriers, while on the other hand, all animals had to be purchased and thus removed from the food chain. This study includes only female turkeys to maximize validity, as the prevalence of GL showed a notably greater variance in the Bronze hen flocks (1.7% to 75.0%) than in the tom flocks (1.7% to 55.0%). Therefore, influences on GL development could potentially be demonstrated [4]. Hens were then taken to the Clinic for Birds and Reptiles at the University of Leipzig and provided with food and water overnight. The next day, all turkeys were euthanized after being stunned (Large Poultry Stunner, Friedr. Dick GmbH & Co., KG, Deizisau, Germany) by subsequent exsanguination through a unilateral neck cut severing the carotid artery and jugular vein. All turkey hens were euthanized for routine diagnostic necropsy.

### 2.2. Data Assessment

Necropsy was conducted following the protocol by Schmidt et al. including the 10-cut procedure protocol implemented by the Food Safety and Inspection Service [29,30]. Pathoanatomical examinations were performed on 360 turkeys. Depending on the occurrence of GL, six hens without and additionally up to six further hens with discoloration were chosen for histopathological, bacteriological, parasitological, and virological examinations. Further examinations were performed on 67 hens at the early and 63 hens at the late fattening stages (in total, 130 hens) (Table S1). Swabs for microbiological examination were taken from the liver, heart, kidney, spleen, lungs, small intestine, shoulder joint, hip joint, knee joint, ankle joint, and the epiphysis of the proximal tibiotarsus. In the case of an ambiguous parasitological result, the diagnosis was confirmed by PCR of the 18S rDNA fragment specific for Eimeria of turkeys and/or for *Tetratrichomonas* (*T.*) *gallinarum* and *Histomonas* (*H.*) *meleagridis* [31,32].

The chymus of the jejunoileum was sequentially diluted 1:10 using phosphate-buffered saline (PBS) for quantifying coliform bacteria as colony-forming units (CFU) (starting with 0.5 g in 4.5 mL PBS). Different volumes of the first two dilutions were cultivated for 24 h at 37 °C on a selective Gassner agar (Sifin, Berlin, Germany), which allows differentiation of lactose fermentation. A Matrix-Assisted Laser Desorption Ionization Time-of-Flight Mass Spectrometer (Bruker, microflex LT mass spectrometer, Bruker Daltonik GmbH Leipzig, Germany) was used to identify bacteria forming colonies on Gassner agar. Analyses for *Brachyspira* (*B.*) sp. were performed randomly at six time points, including at least one examination of each flock. The chymus of the cecum was cultured anaerobically on selective horse blood agar plates designed to detect avian brachyspira as described by Harms et al. [33].

Samples of liver, kidney, spleen, lungs, heart, duodenum, ileum, cecum, and bursa cloacalis were fixed in 4.5% neutral buffered formalin (Formaldehyde-Solution 37%, Merck, Darmstadt, Germany) for at least 24 h. Bone fragments from the femoral head, proximal tibiotarsus, distal tibiotarsus, and proximal tarsometatarsus were decalcified (OSTEOMOLL, Merck KGaA, Darmstadt, Germany) for at least 24 h prior to further processing. Formalin-fixed samples were dehydrated, routinely embedded in paraffin wax, and sectioned at 4 µm. All sections were stained with hematoxylin and eosin for histopathological examination.

Samples for molecular biological examinations were pooled separately for hens with and (if applicable) without GL, despite the assumption that a whole flock would be affected by virus infection. In each case, DNA was extracted from 10 to 15 mg of each pooled sample using the DNeasy Blood & Tissue Kit (QIAGEN, Hilden, Germany) or IndiSpin Pathogen Kit (INDICAL BIOSCIENCE GmbH, Leipzig, Germany). RNA was isolated from 15 to 30 mg of each pooled sample using the RNeasy Mini Kit (QIAGEN, Hilden, Germany). Samples included the trachea and a joint swab for detection of mycoplasma [34]. Samples of liver and duodenal tissue as well as a joint swab were collected for amplification of avian orthoreoviruses [35]. Liver tissue samples were taken to detect the avian hepatitis E virus [36].

For the detection of adenoviruses, spleen and duodenum homogenates were examined [37]. In the case of HEV detection, positive samples were further characterized to determine the virulence and, thus, relevance of the virus. For this purpose, the partial ORF1, E3, and fib knob domains were amplified [38,39]. For amplification of the partial ORF1, E3, and fib knob domains, 2 µL of DNA was mixed with 5 µL of 5X Q5 Reaction Buffer (New England BioLabs, Ipswich, MA, USA), 0.5 µL of dNTPs (final concentration 200 µM; Thermo Fisher Scientific, Waltham, Massachusetts, USA), 1.25 µL of each oligonucleotide primer (final concentration 0.5 µM), and 0.25 µL of Q5 Hot Start High-Fidelity DNA Polymerase (final concentration 0.02 U/µL) (New England BioLabs, Ipswich, MA, USA). The PCR protocol started with activation of the polymerase for 30 s at 98 °C, followed by 40 cycles of denaturation for 10 s at 98 °C, annealing for 15 s at 60 °C, and elongation for 60 s at 72 °C. The reaction ended with a final elongation step lasting 2 min at 72 °C. Subsequently, the PCR product was analyzed by agarose gel electrophoresis. Positive PCR products were purified using the GeneJET PCR Purification Kit (Thermo Fisher Scientific, Waltham, Massachusetts, USA) and subsequently sent to Microsynth Seqlab (Göttingen, Germany) for Sanger sequencing.

Nucleotide sequences were analyzed and edited using the GENtle program (Magnus Manske, University of Cologne, Germany), and comparison was performed using the Basic Local Alignment Search Tool of the National Center for Biotechnology Information (NCBI; https://www.ncbi.nlm.nih.gov/, accessed on 16 May 2022). Phylogenetic analyses and the construction of phylogenetic trees were carried out using the software MEGA X [40]. The evolutionary history was illustrated using the maximum likelihood method and the JTT (Jones-Taylor-Thornton) matrix-based model [41]. For this analysis, the Neighbor-Join and BioNJ algorithms were applied to a matrix of pairwise distances estimated using the JTT model, and then the topology with superior log likelihood value was selected. All sequences of HEV were deposited in NCBI GenBank, with NCBI accession numbers (acc. No.) OM994418, OM994422 to OM994426 for the E3 and fib knob domain genes, and OP171872, OP171876 to OP171880 representing the partial ORF1 gene (Tables S2 and S3).

### 2.3. Statistical Analysis

Statistical analysis was performed with IBM SPSS Statistics for Windows Version 28.0 (IBM SPSS, Armonk, NY, USA) [42]. Tests were implemented to examine infectious agents based on the appearance of GL. Calculations were performed separately for both examination dates to differentiate between the different ages of the hens. A Chi-square test was used to investigate the relationship between two nominal or categorical variables. Fisher’s exact test was utilized instead of the Chi-square test when cell frequencies were below five for 2 × 2 tables. The means of two independent groups were compared using Student’s t-tests for independent samples with a 95% confidence level. The model assumptions of normality and homogeneity of variance were examined by Shapiro-Wilk and Levene’s test. If the data did not follow a normal distribution, the non-parametric Mann-Whitney U test was performed to compare the median values of two samples. A one-way analysis of variance, including a Tukey post-hoc test, was implemented to compare the means of multiple samples. The assumption of normal distribution and homogeneity of variances was determined using the tests described above. A Games-Howell post-hoc test was performed if the data failed to meet the assumption of homogeneity of variances. The non-parametric alternative for the one-way analysis of variance was the Kruskal–Wallis test followed by a post-hoc test. A 95% confidence interval was calculated for the prevalence rates of clinical and pathological findings. For all calculations, results were considered significant if the double-sided *p*-value was equal to or less than 0.05. The *p*-value is supplemented by the specific effect size for the statistical test (d = Cohend’s d, φ = phi coefficient, r = Pearson correlation coefficient).

## 3. Results and Discussion

### 3.1. Gross Pathological and Histopathological Findings

In total, 31 of 343 (9.04%; 95% CI [0.06, 0.12]) Bronze hens (of which 17 turkeys were toms and excluded from further examination) showed GL discoloration. Divided by age, 16 of 184 (8.76%; 95% CI [0.05–0.13]) were affected at the early fattening stage, and 15 of 159 (9.43%; 95% CI: [0.06–0.15]) at the late fattening stage. The prevalence at the individual examination times varied between 0.0 and 68.4%. The most common finding was a focal GL discoloration limited to the caudal margin of the liver. Multifocal or diffuse green coloration appeared rarely. In a previous study, the mean prevalence of GL in organically farmed turkeys was 33.15% (processing batches from nine flocks with 60 Bronze hens each) [4]. Therefore, the mean prevalence for GL is significantly lower in the current study. In addition, examinations in the current study were performed on Bronze hens of a Cartier genetic. This is due to the decreased proportion of organically fattened Kelly BBB turkeys in Germany, which were investigated in the previous study in favor of other turkey genetics. Because both genetics were kept under the same fattening conditions, a comparison of the two studies seems legitimate. The marked discrepancy in GL prevalence between the two studies, however, may be in part due to the different genetics of the turkeys examined. The histopathological examination of the livers revealed the accumulation of bile pigment in 4.62% (95% CI [0.02, 0.09]) of the examined livers and solely at the late stage of fattening. Contrarily, inflammatory reactions consisting of slightly multifocal infiltrates consisting of heterophils, lymphocytes, and plasma cells were seen at both examination times, with a prevalence of 9.23% (95% CI [0.05, 0.15]). There was a significant correlation between GL and the presence of inflammatory cells at both fattening stages (early fattening stage: *p* ≤ 0.05, φ = 0.34; late fattening stage: *p* ≤ 0.05, φ = 0.30), as well as with the accumulation of bile pigment (*p* ≤ 0.001, φ = 0.58) at the late fattening stage (Table 1). Therefore, damage to the hepatic tissue concerning GL can be assumed.

The bodyweight (BW) of hens between their 70th and 75th days of fattening varied from 1.83 kg to 5.41 kg (mean = 3.53 kg, SD = 0.57). Between 120 and 127 days, it ranged from 4.27 kg to 11.91 kg (median = 8.94, variance = 1.60). At the late examination date, differences in BW of more than 2.0 kg between individuals were found in all flocks. Hens with GL had a significantly (*p* ≤ 0.05, d = 0.56) lower mean BW compared to hens without GL in the early fattening period. The discrepancy increased up to 1.45 kg (95% CI [0.36, 2.54]) at the later stage of fattening for hens with GL as opposed to those without (*p* ≤ 0.001, d = 1.19) (Table 1). The reduced body weight in the presence of GL indicates impaired physical development and emphasizes that GL is an indicator of a superordinate pathologic condition.

Over both examination dates, the mean of the relative liver weight was statistically significantly (early fattening stage: *p* ≤ 0.05, d = 0.36; late fattening stage: *p* ≤ 0.01, d = 0.28) higher in hens with GL (Table 2). High relative liver weights as a consequence of liver pathology in cases of GL were already described in the literature [11]. Splenomegaly, defined as follicular hyperplasia, was documented in 5.54% (95% CI [0.035, 0.083]) of hens. Most were seen at the early examination date and predominantly belonged to flock No. 31 in the second trial. However, there was a significant correlation between GL and splenomegaly at the late fattening stage (*p* ≤ 0.001, φ = 0.44). The hens showed a statistically significant (*p* ≤ 0.01, r = 0.35) higher median spleen weight at this stage (Table 1 and Table 2).

Gastrointestinal lesions were seen in 81.54% (95% CI [0.74, 0.88]) of the turkey hens. The most common finding was a slight to mild mucosal and submucosal inflammation consisting of heterophils and lymphocytes. They were occasionally accompanied by intestinal villi thickening and merging. Inflammatory reactions frequently affect more than one intestinal segment. Concerning the targeted organ of this study, a significant relation was proven between the occurrence of GL and catarrhal duodenitis at the early fattening period (*p* ≤ 0.001, φ = 0.47) (Table 2).

A total of 34.69% (95% CI [0.29, 0.40]) of the hens showed skin injuries predominantly located at the snood, and there was no significant correlation for GL. Bilateral footpad dermatitis (FPD) of differing severity was present in every hen. The evaluation method for the footpads is based on a scheme implemented by Mayne and Hocking et al. [43,44]. All lesions were restricted to the upper skin layers, and no alterations of the subcutaneous tissue were found. There was no statistically significant correlation between the severity of FPD and GL (Table 1). Macroscopic alterations of the joints were solely seen in the late fattening phase, with a prevalence of 6.92% (95% CI [0.04, 0.12]). There was a statistically significant correlation between macroscopic bone alterations and GL in the late fattening phase (*p* ≤ 0.001, φ = 0.42) (Table 1). Aseptic bone necrosis was characterized by cell debris and heterophilic and plasmacellular infiltrates accompanied by proliferation of connective tissue and fibrin. It was localized within the epiphyseal growth plate at the proximal tibiotarsus in three cases (2.31%, 95% CI [0.007, 0.06]) and at the distal tibiotarsus in another case (0.77%, 95% CI [0.001, 0.035]). Of those findings, three were seen at the later stage of fattening. Therefore, a significant (*p* ≤ 0.05, φ = 0.40) correlation with GL is given at this stage (Table 2). TOC is defined by green livers associated with lesions of the musculoskeletal system [10,11,45]. Although this study found a correlation between GL and joint/bone lesions, swollen joints did not necessarily indicate GL discoloration. Regarding histological examinations, the relation between aseptic bone necrosis and GL discoloration corresponds to the literature describing TOC as an immune-associated process possibly leading to further lesions [9,11,46]. However, a bacterial infection is not necessarily involved or no longer detectable. This could explain the finding of a single hen with systemic *S. aureus* infection and simultaneous GL discoloration [10,11].

Due to the small sample size (five flocks within two trials), no statistical calculations regarding husbandry and management-associated factors were performed. However, an impact of the housing situation in addition to climatic conditions on several health parameters is likely. Regarding the overall fattening period, total losses ranged from 1.38% to 10.32% (mean = 5.32%, SD = 2.69). A higher median mortality existed in flocks where GL appeared during necropsy. Vaccination against hemorrhagic enteritis virus with DINDORAL SPF (Merial GmbH, Hallbergmoos, Germany) was implemented in flocks No. 21 and 22 in the first examination trial and flock No. 41 in the second examination trial. The time point of vaccination varied between the 21st and the 34th day of fattening (Table S1).

### 3.2. Parasitological Findings

The prevalences of cecal parasites were 45.49% (95% CI [0.37, 0.54]) for *T. gallinarum*, 26.92% (95% CI [0.20, 0.35]) for *Eimeria* (*E.*) *meleagridis*, 23.08% (95% CI [0.17, 0.31]) for *H. meleagridis*, and 3.08% (95% CI [0.01, 0.07]) for *Heterakis* (*H.*) *gallinarum*. No significant relationship between GL and the detection of *E. meleagridis* or *H. gallinarum* could be proven. In contrast, hens with GL significantly more often harbored *T. gallinarum* (*p* ≤ 0.05, φ = 0.27) at the late fattening stage and *H. meleagridis* at the early fattening stage (*p* ≤ 0.001, φ = 0.52) (Table 2). The parasite *T. gallinarum* usually induces latent infections of the ceca without clinically manifest outbreaks. Similar to *H. meleagridis*, the documented association with GL may indicate a general weakening of the affected hens and a higher susceptibility for secondary infections [47,48].

### 3.3. Bacteriological Findings

Extraintestinal bacterial growth was rarely seen in the hens examined. This included one hen at 72 days of fattening with detection of *E. coli* in the spleen, shoulder joint, and knee joint. Despite the presence of osteomyelitis in the distal tibiotarsus, this hen showed no GL. Another hen at 120 days of fattening had a systemic infection from the cultivation of *S. aureus* in her liver, spleen, knee joint, and duodenum. Concurrently, this hen had GL discoloration and inflammatory responses in the liver and spleen on histology.

The detection of *Mycoplasma* sp. succeeded with a prevalence of 9.23% (95% CI [0.05, 0.15]) and predominantly at the late fattening stage. Concerning GL, there was no significant relationship to the detection of mycoplasma. Even though joint/bone lesions predominantly occurred at a later age as well, there was no significant correlation between macroscopic and/or histologic bone lesions and the presence of mycoplasma (Table 2). Most isolates showed high sequence identities with uncultured *Mycoplasma* sp. from geese of ambiguous relevance for turkeys.

Swabs taken from the duodenum revealed *E. coli* in 29.23% (95% CI [0.22, 0.37]) and *Candida* (*C.*) *albicans* in 24.62% (95% CI [0.18, 0.33]) of hens. There was no significant correlation between the detection of *C. albicans* or *E. coli* and GL. The median of the results for quantitative analyses of the commensal *E. coli* in the jejunoileum from each examination time varied between 180 and 110.000 CFU/mL. There was no statistically significant correlation between *E. coli* counts and GL. The most diverse bacterial culture was present in the ceca. Slow-growing thermophilic and microaerobic *Campylobacter* sp. were detected with a total prevalence of 84.62% (95% CI [0.78, 0.90]). Furthermore, *Clostridium* (*C.*) *perfringens* was commonly isolated, with a prevalence of 52.31% (95% CI [0.44, 0.61]). Brachyspira were found with a prevalence of 13.04% (n = 46; 95% CI [0.03, 0.23]). This includes *B. innocens* in 50.00%, *B. pilosicoli* in 16.67%, and brachyspira, which could not be further typified in the remaining positive samples. There was no significant correlation between the detection of *Campylobacter* sp., *C. perfringens*, or *Brachyspira* spp. and GL discoloration (Table 2). It must be noted that four examination trials had problems with clostridial infection during the fattening phase, followed by antimicrobial treatment. Samples were taken at specific time points in which the infection may have resolved despite the persistence of inflammation. Therefore, limited detection of bacterial agents may be explicable.

### 3.4. Virological Findings

In six out of 27 pooled samples from three flocks, HEV was detected irrespective of vaccination status. In addition, *turkey adenovirus 4* (TAdV-4) from two flocks in the late fattening period and *turkey adenovirus 5* (TAdV-5) from two flocks in the early fattening period were detected. Avian orthoreoviruses or *avian hepatitis E virus* RNA were not detected in any pooled sample. Hens from flocks with a positive finding of HEV in the pooled sample revealed GL significantly more often (*p* ≤ 0.001, φ = 0.35) than those without virus detection. Furthermore, hens from positive tested flocks more frequently showed a predominantly slight or moderate catarrhal duodenitis (*p* ≤ 0.001, φ = 0.47) at the early fattening stage (Table 1 and Table 2). In contrast, there was no significant association between the finding of GL and the detection of TAdV-4 or TAdV-5 in the pooled sample at this stage. At the late examination date, hens with HEV-positive samples (7.91 kg, variance = 1.03) showed a significantly (*p* ≤ 0.001, r = 0.59) lower median BW compared to hens without virus detection (9.20 kg, variance = 1.14). Intranuclear inclusion bodies and splenic lymphocytic depletion are characteristics of a pathologically manifest infection with HEV [49]. These alterations were found in five hens in the early fattening period in the second examination trial from flock No. 31. These hens were not vaccinated against HEV but tested virus positive, and one of these hens concurrently had a GL. One key finding of HEV is the eponymous hemorrhagic enteritis, which is primarily localized in the duodenum [49]. In this study, hens with HEV detection only exhibited slight catarrhal duodenitis, indicating a certain effect on intestinal health, nonetheless.

**Figure 1:**
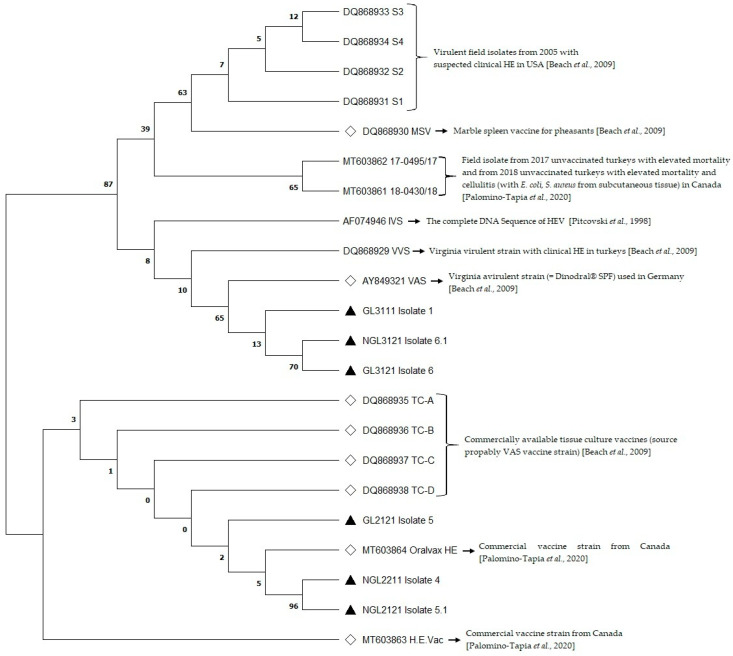
Phylogenetic tree based on concentrated sequences of partial ORF1, E3, and Fiber knob genes (original tree). The evolutionary history was illustrated using the maximum likelihood method and the JTT matrix-based model [41]. The tree with the highest log-likelihood (−1554.26) is indicated. The percentage of trees in which the associated taxa are clustered is indicated next to the branches. For this analysis, the Neighbor-Join and BioNJ algorithms were applied to a matrix of pairwise distances estimated using the JTT model and the topology with the superior log likelihood value. This analysis included 22 amino acid sequences. Six sequences belonging to this study are marked with a black triangle. Vaccine strains are marked with an empty square. The phylogenetic analysis was based on the amino acids of six sequences from this study in comparison to 16 reference sequences from NCBI [17,39,51]. The final data set included a total of 496 positions. The evolutionary analyses were performed in MEGA X [40].

Immunization against HEV is usually part of the routine vaccination regimen for turkeys in Germany. Therefore, we compared the obtained HEV samples with the avirulent turkey HEV strain Virginia (Dindoral^®^ SPF, Merial GmbH, Hallbergmoos, Germany; NCBI acc. No.: AY849321) used for vaccination in Germany [50]. The HEV samples showed high sequence identity (98.98–100.00%) at nucleotide levels at partial ORF1, E3, and fiber knob compared to the listed sequence of the avirulent turkey HEV strain Virginia. This is emphasized by the high sequence identity (98.62–100.00%) at amino acid levels (Tables S2 and S3). The phylogenetic analysis revealed the presence of two main clusters. Isolates from flock 31 cluster close to the Virginia avirulent vaccine strain (Dindoral^®^ SPF) as well as the Virginia virulent strain isolated from turkeys with clinical HE. Isolates from flocks 21 and 22 cluster next to the commercial Oralvax HE vaccine strain listed at NCBI (Figure 1, Table S4). Based on the present phylogenetic analysis, it can be assumed that all isolates cluster close to other field and vaccine strains described in the literature [39,51]. Turkey adenoviruses are known to be widespread in Germany, with particularly avirulent vaccine strains circulating between different turkey flocks. However, knowledge of types and disease conditions is lacking [38,52]. Possible reasons for the contact with the virus could be insufficient cleaning between herds during the rearing or fattening phase or indirect transmission of the virus through employees, insects, rodents, or equipment. Clinical manifestations of HEV infection in flock 31 can be explained by the genetic drift of the circulating HE field and the vaccine strains through which strains gain virulence. However, infection with a HE-isolate does not necessarily lead to a clinical outbreak but can still increase susceptibility to opportunistic pathogens [53]. Therefore, it is necessary to use vaccines that are adapted to the currently circulating strains [15].

Examined turkey flocks were assigned to five subgroups to clarify the potential impact of HEV-vaccination and HEV- or TAdV-5 infection in the early fattening period (flock virus-positive at the first examination time point). The groups were as follows: (a) hens unvaccinated against HEV with detection of TAdV-5; (b) hens unvaccinated against HEV with detection of HEV; (c) HEV-vaccinated hens with detection of HEV; (d) HEV-vaccinated hens without AdV-detection; and (e) hens unvaccinated against HEV without AdV-detection (Table 3). The highest mean mortality rate was seen in the subgroup of HEV-unvaccinated, positive hens. This group also showed the highest prevalence of GL in the early and late fattening periods. Most of the histological bone lesions occurred in groups with either HEV- or TAdV-5-detection for both examination times. At the early fattening period, unvaccinated hens with HEV detection showed the highest prevalence of catarrhal duodenitis. Most of the histological bone lesions were also found during the late fattening period in this group. The mean BW of the hens chosen for further examination did not differ significantly between the five subgroups at the early fattening stage. The same calculations were performed for the relative spleen and liver weights. Although there were no statistically significant differences regarding the relative liver weight, the highest BW proportion at the early fattening stage was found within the group of HEV-unvaccinated and HEV-positive hens. In addition, the mean relative spleen weight at this stage differed significantly between the five groups (*p* ≤ 0.001, η^2^ = 0.32). A Tukey post-hoc analysis revealed a significantly higher relative spleen weight (*p* ≤ 0.05) for unvaccinated hens with HEV detection compared to the other groups. At the early fattening stage, hens of the HEV-unvaccinated but infected subgroup most often harbored *C. perfringens* (100.00% of the hens) and *H. meleagridis* (58.82% of the hens, 95% CI: [0.36–0.79]).

Regarding the detection of HEV, a virally mediated long-term adverse impact can be implied based on the reduced fattening performance at the end of the fattening stage. Overall development of flocks with HEV detection that had not been vaccinated against HEV was worse compared to those that were either HEV-infected but vaccinated or unvaccinated against HEV and AdV-negative. Furthermore, a poor health condition can be presumed due to high prevalences of GL in HEV-unvaccinated but HEV-positive flocks, indicating liver damage; high relative spleen weights, indicating an immunogenic reaction; the presence of inclusion bodies and splenic lymphatic depletion as a pathological side effect of natural HEV infection; and finally, high mortalities. Confirmation is given when assessing the development of HEV-positive and HEV-unvaccinated hens with a high prevalence of GL in addition to joint/bone lesions at the late fattening period. Since the isolates originate from unvaccinated hens infected with a presumably mutated vaccine or field strain, this emphasizes the role of HEV as a predisposing factor for GL and the importance of an adequate vaccination.

## 4. Conclusions

The causative etiology of GL cannot be restricted to one single pathogen. Statistical calculations partially need to be interpreted carefully due to the comparatively low prevalence of GL. Despite the fact that only one hen had a simultaneous *S. aureus* infection, an initial bacterial etiology for the other hens could not be excluded in this study. Furthermore, antimicrobial treatment during the fattening phase could be an explanation for the limited detection of bacterial agents. Bacterial or viral infections determine the general health condition and should be managed adequately to avoid potentially higher GL prevalences. Discoloration should be considered an indicator of a negative impact on individual health status and animal welfare that also entails economic damage. This can be assumed due to decreased weight gain, high relative liver weights, the presence of inflammatory cells in the liver, increased prevalence for catarrhal duodenitis and joint/bone lesions, and a tendency for higher flock mortality in the presence of GL. Furthermore, HEV as an immunosuppressive agent correlated significantly with GL, facilitated secondary infections, increased flock mortality, and led to impaired physical development. This is particularly evident when the hens were not vaccinated against this viral pathogen, which emphasizes the need for proper management of this virus. This study indicates two different pathogeneses for GL with a correlation with immunosuppressive HEV at the early and bone/joint lesions at the late fattening stage. However, the results of this study may be coincidental because other important factors, such as husbandry practices or climate, were not evaluated. Further standardized investigations to determine and evaluate possible infectious risk factors are necessary. This study expanded the knowledge about green liver discoloration in fattening turkeys, nevertheless.

## Figures and Tables

**Table 1:** Comparison of different values for all 343 female Bronze turkey individuals with and without green liver discoloration in the early (70 to 75 days of fattening) and late (120 to 127 days of fattening) fattening stages, respectively. The presented data includes means ($\bar{x}$), medians ($\tilde{x}$), standard deviations (SD), *p*-values, and (if applicable) effect sizes (ES) within the same age group.

Value	70 to 75 Days of Fattening			120 to 127 Days of Fattening		
	Green Liver	No Green Liver		Green Liver	No Green Liver	
	*n* = 16	*n* = 168		*n* = 15	*n* = 144	
	($\bar{x}$)/($\tilde{x}$) ± SD	($\bar{x}$)/($\tilde{x}$) ± SD	*p*-Value/ES	($\bar{x}$)/($\tilde{x}$) ± SD	($\bar{x}$)/($\tilde{x}$) ± SD	*p*-Value/ES
Bodyweight	3.19 ± 0.47	3.57 ± 0.57	0.01/d = 0.56	7.51 ± 1.95	8.97 ± 1.10	≤0.001/d = 1.19
Skin injuries (%)	0.00	17.26	0.08	66.67	55.56	0.41
Splenomegaly (%)	6.25	7.74	1.00	26.67	0.69	≤0.001/φ = 0.44
Macroscopic joint lesions (%)	0.00	0.00		40.00	3.47	≤0.001/φ = 0.42
HEV (%)	93.75	32.74	≤0.001/φ = 0.35	33.33	23.61	0.53
FPD Score	*4.00* * ± 0.27*	*4.00* * ± 0.12*	0.90	*4.00* * ± 0.29*	*4.00* * ± 0.64*	0.48

HEV: Hemorrhagic Enteritis Virus, FPD: Foot pad dermatitis. Data include means and medians (in italic).

**Table 2:** Comparison of different values of the further examined 130 female Bronze turkey individuals with and without green liver discoloration in the early (70 to 75 days of fattening) and late (120 to 127 days of fattening) fattening stages, respectively. The presented data includes means ($\bar{x}$), medians ($\tilde{x}$), standard deviations (SD), *p*-values, and (if applicable) effect sizes (ES) within the same age group.

Value	70 to 75 Days of Fattening			120 to 127 Days of Fattening		
	Green Liver	No Green Liver		Green Liver	No Green Liver	
	*n* = 9	*n* = 58		*n* = 15	*n* = 48	
	($\bar{x}$)/($\tilde{x}$) ± SD	($\bar{x}$)/($\tilde{x}$) ± SD	*p*-Value/ES	($\bar{x}$)/($\tilde{x}$) ± SD	($\bar{x}$)/($\tilde{x}$) ± SD	*p*-Value/ES
Rel. weight spleen (%)	*0.11* * ± 0.00*	*0.08* * ± 0.00*	0.12	*0.09* * ± 0.00*	*0.06* * ± 0.00*	0.004/ r = 0.35
Rel. weight liver (%)	2.25 ± 0.55	1.91 ± 0.33	0.01/ d = 0.36	1.67 ± 0.51	1.26 ± 0.15	0.004/ d = 0.28
Inflammatory cells liver	22.22	1.72	0.045/ φ = 0.34	33.33	8.33	0.03/ φ = 0.30
Macroscopic joint lesions (%)	0.00	0.00		40.00	3.47	≤0.001/ φ = 0.42
Bile pigment liver	0.00	0.00	-	40.00	0.00	≤0.001/ φ = 0.58
Lymphocytic depletion spleen	11.11	10.34	1.00	0.00	0.00	-
Aseptic bone necrosis	0.00	1.72	1.00	20.00	0.00	0.01/ φ = 0.40
Catarrhal duodenitis	66.67	12.07	≤0.001/ φ = 0.47	26.67	10.42	0.20
Catarrhal jejunitis	22.22	46.55	0.28	33.33	37.50	0.77
Catarrhal typhlitis	22.22	62.07	0.03/ φ = 0.27	73.33	81.25	0.49
Qual. *Escherichia coli* duodenum	11.11	27.59	0.43	46.67	29.17	0.21
Quan. *Escherichia coli* duodenum (CFU)	*4000* * ± 1.8 × 10^8^*	*3950* * ± 5.6 × 10^11^*	0.41	*2.800* * ± 1.1 × 10^11^*	*1350* * ± 2.5 × 10^10^*	0.68
*Candida albicans* duodenum	66.67	32.76	0.07	6.67	12.50	1.00
*Staphylococcus aureus* extraintestinal	0.00	0.00	-	6.67	0.00	0.24
*Escherichia coli* extraintestinal	0.00	6.70	1.00	6.67	2.08	0.42
*Campylobacter* sp.	77.78	68.97	0.71	100.00	100.00	-
*Clostridium perfringens*	88.89	62.07	0.15	40.00	37.50	1.00
*Brachyspira* sp.^1^	16.67	0.00	0.27	0.00	27.78	0.28
*Mycoplasma* sp.	0.00	3.45	1.00	6.67	18.75	0.43
*Tetratrichomonas gallinarum*	77.78	43.10	0.08	66.67	35.43	0.03/ φ = 0.27
*Histomonas meleagridis*	77.78	13.79	≤0.001/ φ = 0.52	13.33	27.08	0.49
*Heterakis gallinarum*	0.00	0.00	-	0.00	8.33	0.56
*Eimeria meleagridis*	0.00	29.31	0.10	26.67	29.17	1.00

rel. = relative; qual. = qualitative; quan. = quantitative. ^1^ Investigations were performed randomly at six examination points. Data include means and medians (in italic).

**Table 3:** Presentation of different values for flocks or selected hens of (a) hens unvaccinated against HEV with detection of TAdV-5, (b) hens unvaccinated against HEV with detection of HEV, (c) HEV-vaccinated hens with the detection of HEV, (d) HEV-vaccinated hens without AdV-detection, and (e) hens unvaccinated against HEV without AdV-detection. The presented data includes means ($\bar{x}$), except for the relative liver weight, which is given as medians ($\tilde{x}$).

Group	a	b	c	d	e
**Value—Flocks**					
***n***	37	39	31	20	57
Mortality (%)	5.54	9.15	3.04	7.08	3.57
Green livers ^1^ (%)	0.00	35.90	3.20	0.00	1.80
Green livers ^2^ (%)	12.80	20.00	5.00	10.00	5.00
**Value—Selected hens**					
***n***	12	17	13	6	19
BW ^1^ (kg)	3.33	3.57	3.69	3.42	3.42
BW ^2^ (kg)	7.11	8.03	10.29	7.96	9.41
Rel. weight spleen ^1^ (%)	0.07	0.12	0.08	0.09	0.09
Rel. weigh liver ^1^ (%)	*2.04*	*2.07*	*1.84*	*1.73*	*1.95*
Bone lesions ^1,3^ (%)	16.70	5.90	30.80	0.00	10.50
Bone lesions ^2,3^ (%)	5.90	20.00	14.30	12.50	0.00
Catarrhal duodenitis ^1^ (%)	0.00	64.71	7.69	16.67	0.00
*Clostridium perfringens*^1^ (%)	50.00	100.00	53.85	50.00	57.89
*Histomonas meleagridis*^1^ (%)	25.00	58.82	0.00	0.00	10.53

^1^ first examination (70th–75th day of fattening); ^2^ second examination (120th–127th day of fattening); ^3^ histological bone lesions; Data include means and medians (in italic).

## Data Availability

The data presented in this review is available on request from the corresponding author.

## Supplementary Materials

The following supporting information can be downloaded at: https://www.mdpi.com/article/10.3390/ani13050918/s1. Table S1: Anamnestic data of Bronze turkey hens of a Cartier genetic from five organic turkey farms (two trials each); Table S2: Nucleotide (nt) and amino acid (aa) sequence identities of detected hemorrhagic enteritis virus at the early fattening period in organic fattened turkeys compared to virulence genes E3 (nt 21247–22116 [aa AAX51187.2]) and Fiber (nt 22519-23883 [aa AAX51189.1]) of avirulent turkey hemorrhagic enteritis virus strain Virginia (NCBI acc. No.: AY849321.1) in %; Table S3: Nucleotide (nt) and amino acid (aa) sequence identities of detected hemorrhagic enteritis virus at the early fattening period in organic fattened turkeys compared to virulence gene ORF 1 (nt 313–1953 [aa AAX51169.2]) of avirulent turkey hemorrhagic enteritis virus strain Virginia (NCBI acc. No.: AY849321.1) in %; Table S4: List of samples used for comparative analysis in this study with NCBI accession numbers (acc. no). The table includes several (virulent) field samples as well as commercial vaccine strains against hemorrhagic enteritis (HE). Sorted by authors and publication journals [17,39,51].
