# Supplementary material for: An Explorative Study of the Causal Pathogenesis of Green Liver Discoloration in Organically Reared Female Bronze Turkeys (Meleagris gallopavo) Considering the Infectious Risk Factors
Source: Animals (Basel). 2023 Mar 3;13(5):918. doi: 10.3390/ani13050918 (PMC10000099; doi:10.3390/ani13050918)
Supplement: Supplementary file 1 [file animals-13-00918-s001.zip › animals-2206748-supplementary.pdf]

Table S1. Anamnestic data of Bronze turkey hens of a Cartier genetic from five organic turkey farms (two trials each)

**Flock no.**	**11**		**21**		**22**		**31**		**41**	
**Trial**	**1**	**2**	**1**	**2**	**1**	**2**	**1**	**2**	**1**	**2**
Total count[1]	18/20	20/20	20/20	11/20	20/20	20/20	19/[2]	20/20	19/[2]	17/19
Further investigated[1]	6/6	7/8	6/8	7/6	6/8	6/6	10/[2]	7/10	6/[2]	6/11
Green livers during examinations	no	yes	yes	yes	yes	no	yes	yes	no	yes
access to free-range area	yes	to day 115	yes	no	no	yes	yes	yes	yes	yes
total mortality (%)	4.66	4.67	7.08	1.80	4.28	5.47	10.32	7.97	1.38	5.60
vaccinations	Er, ND	Er, ND	Ec, HEV, ND, TRT	Ec, HEV, ND, TRT	Ec, HEV, ND, TRT	Ec, HEV, ND, TRT	ND	ND	Ec, Er, ND, ORT, Pas, Sta	Ec, Er, HEV, ND, ORT, Pas, TRT, Sta
coccidiosis	no	no	yes	no	yes	no	yes	yes	no	yes
coccidiostats	no	no	yes[3]	yes[3]	yes[3]	yes[3]	yes[3]	yes[3,4]	no	yes[3]
clostridiosis	yes	no	yes	no	no	no	yes	yes	no	yes
antibiotics	no	no	yes[5,6]	no	no	no	yes[5]	yes[5]	no	yes[5]

Ec = *Escherichia coli*, Er = *Erysipelothrix rhusiopathiae*, HEV = hemorrhagic enteritis virus, ND = Newcastle Disease, ORT = *Ornithobacterium rhinotracheale*, Pas = *Pasteurella* multocida., Sta = *Staphylococcus aureus*, TRT = Turkey rhinotracheitis

[1]Examinations 1/2

[2]no examination

[3]Toltrazuril (Toltra-K 25 mg/ml, Bela-Pharm GmbH &. Co. KG, Vechta, Germany)

[4]Amprolium (Eimeryl 200 mg/ml, Virbac Tierarzneimittel GmbH, Bad Oldesloe, Germany)

[5]Amoxicillin Solamocta 697 mg/g, (Dechra Veterinary Products Deutschland GmbH, Aulendorf, Germany)

[6]Tylosin (Pharmasin 100.00 %, Huvepharma N.V., Antwerp, Belgium)

Table S2. Nucleotide (nt) and amino acid (aa) sequence identities of detected hemorrhagic enteritis virus at the early fattening period in organic fattened turkeys compared to virulence genes E3 (nt 21247 – 22116 [aa AAX51187.2]) and Fiber (nt 22519-23883 [aa AAX51189.1]) of avirulent turkey hemorrhagic enteritis virus strain Virginia (NCBI acc. no.: AY849321.1) in %.

NCBI acc. no	Sequence ID	Isolate	E3 (nt)	E3 (aa)	Fiber (nt)	Fiber (aa)
OM994418	GL3111	Isolate 1	99.54	98.62	99.63	99.12
OM994422	NGL2211	Isolate 4	99.77	99.31	100.00	100.00
OM994423	NGL2121	Isolate 5.1	99.77	99.31	100.00	100.00
OM994424	GL2121	Isolate 5	99.77	99.31	100.00	100.00
OM994425	NGL3121	Isolate 6.1	99.89	99.65	99.36	99.34
OM994426	GL3121	Isolate 6	99.89	99.65	99.71	99.34

Table S3. Nucleotide (nt) and amino acid (aa) sequence identities of detected hemorrhagic enteritis virus at the early fattening period in organic fattened turkeys compared to virulence gene ORF 1 (nt 313 – 1953 [aa AAX51169.2]) of avirulent turkey hemorrhagic enteritis virus strain Virginia (NCBI acc. no.: AY849321.1) in %.

NCBI acc. no	Sequence ID	Isolate	ORF1 (nt)	ORF1 (aa)
OP171872	GL3111	Isolate 1	99.86	100.00
OP171876	NGL2211	Isolate 4	99.44	98.79
OP171877	NGL2121	Isolate 5.1	99.44	98.79
OP171878	GL2121	Isolate 5	99.51	99.20
OP171879	NGL3121	Isolate 6.1	99.72	99.80
OP171880	GL3121	Isolate 6	99.72	99.80

Table S4. List of samples used for comparative analysis in this study with NCBI accession numbers (acc. no). The table includes several (virulent) field samples as well as commercial vaccine strains against hemorrhagic enteritis (HE). Sorted by authors and publication journals.

NCBI acc. no	Isolate designation	Source	Type	Year	Clinical Case	Publication
OP171872	Isolate 1	Turkey spleen	Field	2020	High mortality, high relative spleen weight	Present study
OP171876	Isolate 4	Turkey spleen	Field	2021	-	
OP171877	Isolate 5.1	Turkey spleen	Field	2021	-	
OP171878	Isolate 5	Turkey spleen	Field	2021	-	
OP171879	Isolate 6.1	Turkey spleen	Field	2021	High mortality, high relative spleen weight, intranuclear inclusion bodies and lymphocytic depletion in spleens	
OP171880	Isolate 6	Turkey spleen	Field	2021		
AY849321	Virginia avirulent strain (VAS)	Pheasant spleen	Vaccine	2004	From pheasants with enlarged spleens	[51]
DQ868931	Virulent field isolate 1 (S-1)	Virginia field isolate	Field	2005	Suspected clinical HE	
DQ868932	Virulent field isolate 2 (S-2)	Virginia field isolate	Field	2005	Suspected clinical HE	
DQ868933	Virulent field isolate 3 (S-3)	Virginia field isolate	Field	2005	Suspected clinical HE	
DQ868934	Virulent field isolate 4 (S-4)	Virginia field isolate	Field	2005	Suspected clinical HE	
DQ868935	Tissue culture vaccine A (TC-A)	Turkey spleen	Vaccine	2005	-	
DQ868936	Tissue culture vaccine B (TC-B)	Commercial	Vaccine	1991	-	
DQ868937	Tissue culture vaccine C (TC-C)	Commercial	Vaccine	1992	-	

DQ868938	Tissue culture vaccine D (TC-D)	Commercial	Vaccine	1992	-	
DQ868929	Virginia virulent strain (VVS)	Turkey spleen	Field	1996	Clinical HE	
DQ868930	Marble spleen vaccine (MSV)	Turkey litter	Vaccine	1987	-	
MT603863	H.E.Vac	Commercial	Vaccine	2020	-	[39]
MT603864	Oralvax HE	Commercial	Vaccine	2020	-	
MT603861	18-0430-AB-2018	Turkey spleen	Field	2018	Increased Mortality-Cellulitis	
MT603862	17-0495-ON-2017	Turkey spleen	Field	2017	Increased Mortality-Surveillance	
AF074946	Israel virulent strain (IVS)	Israel field isolate	Field	1998	Unknown	[17]
